# Functional Impairment of Human Myeloid Dendritic Cells during *Schistosoma haematobium* Infection

**DOI:** 10.1371/journal.pntd.0000667

**Published:** 2010-04-20

**Authors:** Bart Everts, Ayola A. Adegnika, Yvonne C. M. Kruize, Hermelijn H. Smits, Peter G. Kremsner, Maria Yazdanbakhsh

**Affiliations:** 1 Department of Parasitology, Leiden University Medical Centre, Leiden, The Netherlands; 2 Institute of Tropical Medicine, University of Tübingen, Tübingen, Germany; 3 Medical Research Unit, Albert Schweitzer Hospital, Lambaréné, Gabon; McGill University, Canada

## Abstract

Chronic *Schistosoma* infection is often characterized by a state of T cell hyporesponsiveness of the host. Suppression of dendritic cell (DC) function could be one of the mechanisms underlying this phenomenon, since *Schistosoma* antigens are potent modulators of dendritic cell function *in vitro*. Yet, it remains to be established whether DC function is modulated during chronic human *Schistosoma* infection *in vivo*. To address this question, the effect of *Schistosoma haematobium* infection on the function of human blood DC was evaluated. We found that plasmacytoid (pDC) and myeloid DC (mDC) from infected subjects were present at lower frequencies in peripheral blood and that mDC displayed lower expression levels of HLA-DR compared to those from uninfected individuals. Furthermore, mDC from infected subjects, but not pDC, were found to have a reduced capacity to respond to TLR ligands, as determined by MAPK signaling, cytokine production and expression of maturation markers. Moreover, the T cell activating capacity of TLR-matured mDC from infected subjects was lower, likely as a result of reduced HLA-DR expression. Collectively these data show that *S. haematobium* infection is associated with functional impairment of human DC function *in vivo* and provide new insights into the underlying mechanisms of T cell hyporesponsiveness during chronic schistosomiasis.

## Author Summary

A key feature of schistosomiasis, as well as of other systemic helminth infections, is their chronic nature. This reflects the successful evasion and suppression of host immune responses by the parasites. One of the mechanisms that could underlie this phenomenon is modulation of the dendritic cells (DC), which play a central role in initiation and control of T cell responses. Although several *in vitro* studies have documented the modulatory capacity of *Schistosoma* antigens on DC function, it is still unclear what effect schistosomiasis has on human DC function *in vivo*. To address this question, we isolated the two main DC subsets present in peripheral blood from infected and uninfected individuals living in a *Schistosoma*-endemic area in central Africa, and found that specifically the myeloid DC from *Schistosoma*-infected subjects displayed an impaired capacity to respond to Toll-like receptor ligands and to drive T cell responses. These findings provide new insights into how schistosomiasis suppresses host immune responses, which can be exploited for new strategies to enhance immunity against the parasites.

## Introduction

Chronic parasitic helminth infections are generally associated with a T helper 2 (Th2)-like immunological profile as well as immune hyporesponsiveness [1], [2]. The latter characteristic is probably the result of immune evasion strategies that these pathogens have developed to ensure long term survival within their hosts and to allow continued transmission. Also chronic infections with blood-dwelling trematodes of the genus *Schistosoma* are well known for their capacity to suppress immune responses, which is not only reflected in negative regulation of parasite specific immune responses but also by attenuation of effector responses to third party antigens, including allergens [3]–[5], and other pathogens [6], [7].

Dendritic cells (DC) are the most important antigen presenting cells (APC) that initiate immune responses and as such play a central role in the development and regulation of protective immunity against invading pathogens as well as tolerance under homeostatic conditions [8], [9]. In peripheral blood, two major DC subsets can be identified: the CD11c^+^ myeloid DC (mDC) and CD123^+^ plasmacytoid DC (pDC) [10]. Both are immature DC that are in the process of migration to their target sites. It is believed that these subsets perform different functions in both innate and adaptive immune responses. Human mDC express Toll-like receptors (TLR)-2, -4 and -8 and are thought to preferentially induce T cell responses to invading pathogens. On the other hand, human pDC have a different but complementary TLR expression profile that includes TLR-7 and -9 and are believed to play an important role in innate anti-viral responses as well as in induction of tolerance against self-antigens [11], [12].

One of the main characteristics of immune hyporesponsivess during chronic schistosomiasis is the impairment of effector T cell responses, a process in which T cell anergy and regulatory T cells have been proposed to play an important role [13], [14]. Given that DC are central players in the priming and regulation of T cell responses, suppression of these host immune responses by schistosomes through modulation of DC function would seem plausible. Indeed, studies have demonstrated that host derived anti-inflammatory mediators, such as IL-10 [15], which is elevated during chronic schistosomiasis [5], [16], [17], as well as *Schistosoma*-derived molecules [18], [19] have the potential to modulate DC function and its T cell priming capacity *in vitro*. However, it remains to be established whether human DC function is actually modulated *in vivo* during chronic schistosomiasis.

To address this question, we evaluated the function of blood DC in a cross-sectional study in Gabon, a region endemic for *Schistosoma haematobium* [5]. We find that mDC isolated from infected subjects have an impaired capacity to respond to TLR ligands and to prime T cell responses. In addition, our data indicate that the former observation is due to a dampened pro-inflammatory signaling rather than lower TLR expression, and that the latter finding originates from a reduced expression of HLA-DR. Collectively, the data show a functional impairment of blood mDC during chronic *S. haematobium* infection and shed new light on the mechanisms underlying immune hyporesponsivess during chronic helminth infections.

## Materials and Methods

### Ethics statement

The study was approved by the Comité d'Ethique Régional Independent de Lambaréné (CERIL). Written informed consent was obtained from all subjects participating in the study.

### Study population

Venous blood was obtained from 43 young adults (mean age 25,3±5,8 years) living in or in the vicinity of Lambaréné, Gabon, a semi-urban municipality and an area in which *Schistosoma haematobium* infection is endemic [5]. Infection with *S. haematobium* was determined by passing 10 ml urine through 12 µm diameter filter. From every subject two independent urine screenings were performed to increase accuracy. The subjects were grouped into an infected group and non-infected group, which were sex and age matched. Furthermore, subjects were screened for other parasitic blood infections, including malaria and filariasis, as determined by microscopic analysis of Giemsa-stained blood smears (Table 1). For HLA-DR and/or CD80 neutralization experiments in the DC-T cell cocultures, mDC were isolated from venous blood from healthy European subjects.

**Table 1:** Study population.

	*S. haematobium* infected n = 23	Uninfected n = 20	p value
Area	Gabon, Lambaréné & vicinity	Gabon, Lambaréné	
Male/female	17/6	17/3	0.71^♦^
Mean age in years (range)	25.2 (17–39)	27.2 (20–37)	0.27
Mean egg counts (range)Δ	267 (1–5000)	0	ND
Subjects with co-infection			
Malaria*	1	0	ND
Microfilaria*^,#^	11	5	0.22^♦^

**Δ:** , based eggs present in 10 ml urine trapped by a 12 µm filter; *, based on Giemsa-stained whole blood smear; ^#^, based on presence of microfilaria in cell cultures; ^♦^, X^2^ analysis with Fisher's exact test; ND, not determined.

### Blood DC isolation

PBMCs were isolated by Ficoll-Hypaque density gradient centrifugation from 40 ml of heparinized venous blood. Myeloid DC (mDC; BDCA-1+) were isolated by CD19-negative selection, followed by CD1c-positive selection using MACS and BDCA-1 Dendritic Cell Isolation Kit (Miltenyi Biotec). Subsequently, to maximize cell yield plasmacytoid DC (pDC; BDCA4^+^/CD123^+^) were isolated from the flowthrough of the mDC isolation by using and BDCA-4 Dendritic Cell Isolation Kit (Miltenyi Biotec). Purity based on BDCA-1/CD11c and BDCA-4/CD123 expression for myeloid and plasmacytoid DCs, respectively, was routinely more than 90%.

### Characterization of DC populations in peripheral blood

For immunophenotyping and determination of frequencies of mDC and pDC present in peripheral blood, part of freshly isolated PBMCs were fixed using a ‘fixation and dead cell discrimination kit’ (Miltenyi Biotec), according to the manufacturers recommendations. Subsequently, cells were washed in 0,5%BSA/PBS and stained for 30 minutes at 4°C with anti-CD19-Pacific blue (Biolegend), anti-CD14-Pacific blue (Biolegend), anti-HLA-DR-APC/cy7 (Biolegend), anti-BDCA-1-APC (Miltenyi Biotec) and anti-BDCA-2-biotin/streptavidin-Qdot526 (both eBioscience) in combination with either anti-CD80-PE/Cy5 (BD) and anti-TLR4-FITC (BD) or anti-CD86-FITC (BD) and anti-CCR7-PE/Cy7 (BD). As gating strategy, blood DC were selected as the CD14^−^/CD19^−^/HLA-DR^+^ population, followed by gating on BDCA-1 and BDCA-2 positive cells as selective markers for mDC and pDC, respectively. Frequencies as well as expression levels of TLR4, CD80, CD86, HLA-DR, CCR7 on these subpopulations were determined.

### Blood DC culture

Freshly isolated DC (2×10^4^ cells/well in 200 µl) were cultured in complete RPMI 1640 medium containing 10% FCS, 100 U/ml penicillin, and 100 µg/ml streptomycin and supplemented with 500 U/ml GM-CSF or 10 ng/m IL-3 (both Strathmann, Germany) for mDC and pDC respectively. DC were stimulated with 100 ng/ml LPS (ultrapure, *E. coli* 0111 B4 strain), 1 µg/ml R848, or 1 µg/ml CpG-B 2006 (all Invivogen). After 40 h supernatants and cells were harvested. For analysis of surface marker expression of TLR stimulated DC, the cells were fixed in 2% formaldehyde (Sigma-Aldrich) for 15 minutes and, after two washing steps in 0,5%BSA/PBS, stained for 30 minutes at 4°C with a combination of either anti-HLA-DR-PerCP, anti-CD80-PE, anti-CD86-FITC and anti-CD40-APC or anti-PD-L1-APC, anti-ICOS-L-PE and anti-CCR7-FITC (all BD).

### MAPK assay

Freshly isolated DCs (2×10^4^ cells/well in 200 µl) were seeded to rest for 24 h in 96-well round bottom plates before stimulation with LPS. After 20 or 60 minutes cells were fixed for 10 minutes with 4% ultrapure formaldehyde (Polysciences) directly in the plate. Cells were harvested and washed twice in PBS/0.5% BSA. Subsequently, the DCs were permeabilized in 700 µl ice-cold 90% methanol in PBS and stored at −80°C until analysis. For intracellular staining of phosphorylated MAPK, fixed permeabilized DC were washed twice in PBS/0.5%BSA and subsequently stained with anti-phospho-p44/42 MAPK AF-488 (T202/Y204) and anti-phospho-p38 MAPK AF-647 (T180/Y182), (both Cell Signalling Technology) for 2 hours at room temperature in the dark.

### mDC coculture with naive T helper cells

5×10^3^ LPS-matured mDC were cocultured with 2×10^4^ allogeneic naive T helper cells in 96-well flat-bottom plates in 200 µl (Corning). Naive T helper cells were purified from a single European donor using the human CD4+/CD45RO- column kit (R&D, Minneapolis, MN). In some experiments cocultures were performed in the presence of 10 µg/ml IgG1, blocking mAbs against HLA-DR (varying concentrations, clone L243, Biolegend) or CD80 (5 µg/ml, clone 37711) (both R&D systems). After 6 days supernatants were harvested for determination of cytokine levels. In addition, T cells were counted with a counting chamber. For intracellular cytokine analysis T cells were restimulated with 200 ng/ml PMA and 2 µg/ml ionomycin in the presence of 10 µg/ml brefeldin A (all Sigma-Aldrich) during 6 h, followed by fixation in 4% formaldehyde (Sigma-Alldrich) for 15 minutes at RT. After washing in 0.5% saponin/PBS, T cells were stained for 30 minutes in 0.5% saponin/PBS with anti-IL4-PE, anti-IFNγ-FITC, anti-TNF-α-biotin, anti-IL-10-APC (all BD Biosciences) followed by a second incubation with streptavidin-PerCP (eBioscience) at RT. In addition, T cells were stained for activation markers CD25-APC and HLA-DR-FITC (both BD).

### Cytokine analysis

Cytokine levels in supernatants from 40 h stimulated DC and 6 d T cells cultures were determined using Luminex-100 cytometer (Luminex Corporation, TX, USA). DC supernatants were analysed simultaneously for IL-1β, IL-6, IL-10, IL-12, TNF-α and IFN-α, while in T cell supernatants levels of IL-10, TNF-α, IL-4, IL-5, IL-13 and IFN-γ were determined. Buffer reagent and Luminex cytokine kits (Biosource, CA, USA) for cytokine analysis were used according to the manufacturers' recommendations. Samples with concentrations below the detection limit, as determined by the provided standards, were given half the value of this threshold.

### Flow cytometric analysis

FACS experiments were performed on a Becton Dickinson FACSCalibur flowcytometer (BD) with CellQuest software (BD), except for the direct characterization of blood DCs in PBMCs, which was performed on a FACSCanto II (BD) using FACSDiva software. For the latter analysis, Fluoresence-minus-one (FMOs) was taken along as controls. FACS data were analysed using FlowJO software (Treestar, USA).

### Statistical analysis

Data were analysed using SPSS (v14.0) and GraphPad Prism (v5). For multiple comparisons, the Kruskal-Wallis H nonparametric test was applied. Statistical difference between two groups was determined by applying the Mann-Whitney *U* nonparametric test. A paired *t* test was used to show the effect of treatment with specific blocking or stimulating abs on T cell function. Differences were considered significant when P-values were below 0.05.

## Results

### *S*. *haematobium* infected subjects have lower frequencies of mDC and pDC in peripheral blood

**Figure 1:**
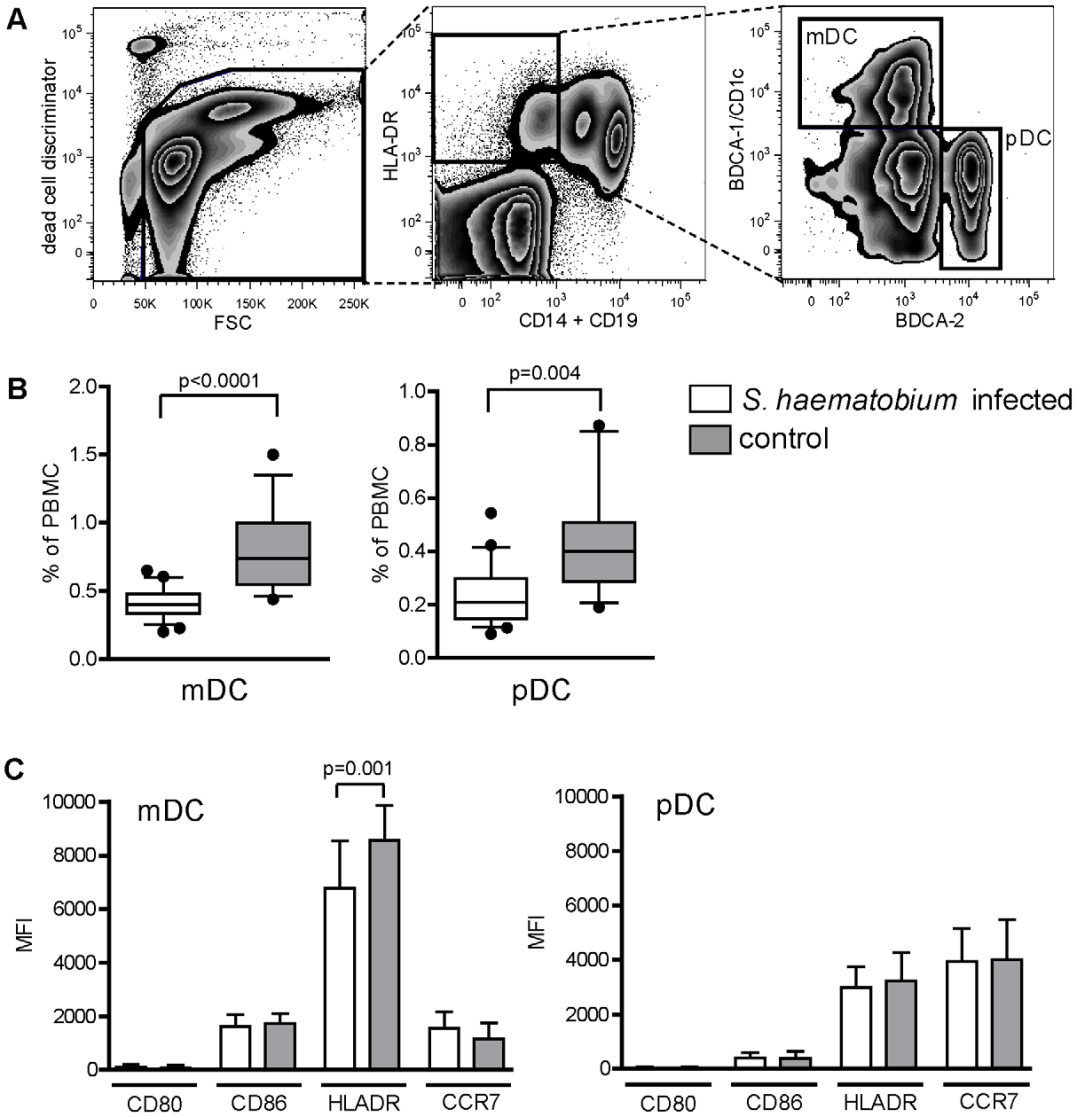
Reduced frequencies of mDC and pDC in blood from *S. haematobium*-infected subjects. (A) Blood DC were identified in fixed PBMC as HLA-DR^+^/CD14^−^/CD19^−^ cells and subsequently subdivided into mDC and pDC on the basis of positive staining for BDCA-1 and BDCA-2, respectively. Data from one representative donor is shown. (B) Frequencies of blood DC subsets in total PBMC (C) and their surface expression of HLADR, CD80, CD86 and CCR7 was determined by following the gating strategy shown in (A). (B) Box and whiskers with 10–90% percentile are shown. (C) Bars represent mean + SD. (B+C) Each group represents data from 20 donors.

To study the effect of *S. haematobium* infection on human dendritic cell phenotype and function, we characterized the two major dendritic cell subsets present in peripheral blood, CD11c/BDCA1^+^ myeloid DC (mDC) and CD123^+^/BDCA2^+^ plasmacytoid DC (pDC) [11], in 23 infected and 20 uninfected individuals recruited from a *S. haematobium* endemic area in Gabon (for characteristics of the study population, see Table 1). The frequencies of mDC and pDC were determined in peripheral blood mononuclear cells (PBMC) isolated from infected and uninfected subjects, by gating on HLA-DR^+^/BDCA1^+^/CD14^−^/CD19^−^ and HLA-DR^+^/BDCA2^+^/CD14^−^/CD19^−^ populations, respectively (Fig. 1A). Both mDC and pDC frequencies were significantly reduced in the infected group compared to the uninfected subjects (Fig. 1B). Of note, the infected individuals were found to have higher PBMC counts (1.64*10^6^/ml ±0.13) compared to the uninfected subjects (1.26*10^6^/ml ±0.11) (p = 0,04). Nonetheless, the absolute numbers of both pDC and mDC/ml blood were still lower in the infected individuals compared to the uninfected individuals (pDC from infected: 0.37*10^4^/ml ±0.04 versus uninfected 0.61*10^4^/ml blood ±0.11, p = 0,02, and mDC from infected: 0.66*10^4^/ml blood ±0.06 versus uninfected: 1.05*10^4^/ml ±0.16, p = 0,01). In addition, to determine whether the activation status of the DC subsets differs between infected and uninfected subjects surface expression of HLA-DR, CD80, CD86, CD40 and CCR7 was analysed. In particular HLA-DR expression on mDC was significantly lower in the infected subjects (Fig. 1C), indicating that mDC are phenotypically different during infection. The expression of the other markers was not different between the groups.

### mDC, but not pDC, from S. *haematobium* infected subjects have reduced responses to TLR ligands

**Figure 2:**
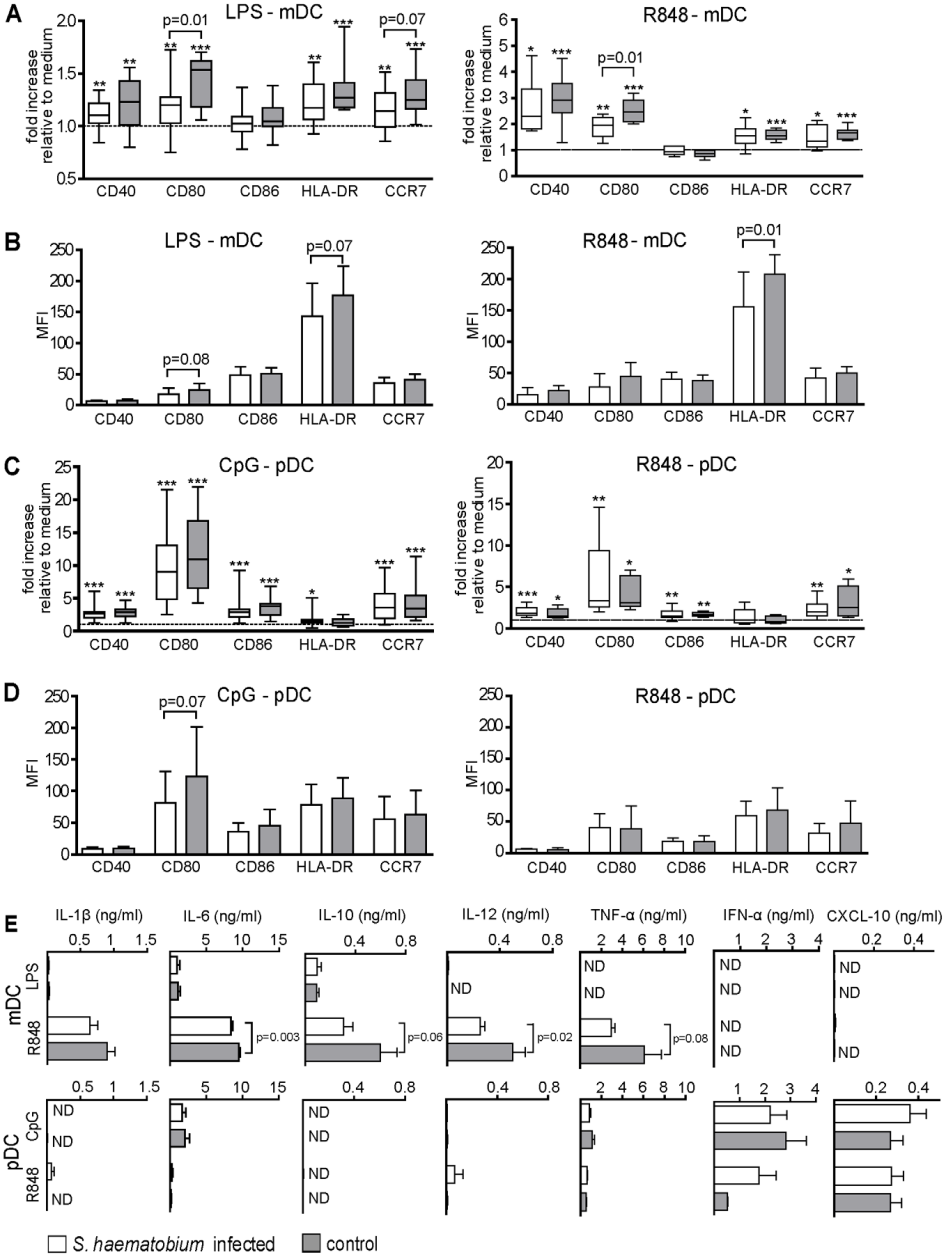
mDC, but not pDC, from *S. haematobium*-infected subjects have impaired TLR responses. Isolated blood DC were stimulated with 100 ng/ml LPS (mDC), 1 µg/ml R848 (mDC & pDC) or 1 µg/ml CpG (pDC) for 40 h. (A–D) LPS- and R848-matured mDC and CpG and R848 stimulated pDC were analysed for maturation marker expression by FACS. Expression of surface marker is either shown (A+C) as fold increase relative to medium or (B+D) as absolute mean fluorescence intensity. (E) Cytokines levels in 40 h culture supernatants from TLR-stimulated DC were determined by multiplex Luminex. Values represent cytokines concentration from which medium control cytokines levels have been subtracted. (A+C) box plots represent 25–75 percentile range with error bars showing minimum to maximum. *, p<0,05; **, p<0,01; ***, p<0,001 for significant differences in fold increase in maturation marker expression relative to the medium-stimulated DC. (B, D and E) Bars represent mean + SD. Each group represents data from 20 donors, except for (E) in which R848-stimulated pDC cytokine data are represented by 4 and 5 donors for the infected and un-infected group, respectively. ND: not detectable.

Schistosomal antigens have been shown to harbour the capacity to modulate and suppress TLR-induced activation of *in vitro* generated DC [18]–[20]. To address whether TLR-mediated activation of human blood DC during *Schistosoma* infection is also affected, mDC and pDC were isolated from peripheral blood of infected and uninfected individuals. Subsequently mDC were stimulated by LPS, a TLR4 ligand and well known myeloid DC activator, while pDC were stimulated by TLR9 ligand CpG [21]. In addition, both DC subsets were stimulated with R848 (TLR7/8 ligand), as this is a TLR ligand that can both activate mDC and pDC. To characterize the responses of the DC subsets to the different TLR ligands, the surface expression of maturation markers was determined. As expected, expression of all markers (CD40, CD80, HLA-DR and CCR7) on mDC was increased in response to LPS as well as R848, except for CD86 (Fig. 2A: fold increase compared to medium cultured mDC). Interestingly, the fold increase in expression of CD80 in response to LPS and R848 and CCR7 in response to LPS was significantly lower in the cultured mDC derived from the *Schistosoma*-infected group compared to the uninfected group (Fig. 2A). The mean fluorescence intensity (MFI) of CD80 and HLA-DR tended to be lower on mDC derived from *S. haematobium*-infected subjects after LPS stimulation and significantly lower for HLA-DR in response to R848 (Fig. 2B). CpG and R848 induced an increase in surface markers on pDC, but with no significant differences between the two groups (Fig. 2C), although in absolute expression levels, there was a tendency towards a lower CD80 expression in the infected group following CpG stimulation (Fig. 2D).

To further characterize the TLR responsiveness of the blood DC, several cytokines were analysed in supernatants (Fig. 2E). LPS-stimulated mDC produced only very low levels of IL-10 and IL-6, whereas R848 induced much more and higher cytokine responses in mDC. Interestingly, mDC from infected subjects had a significantly impaired IL-12 and IL-6 production in response to R848 relative to mDC isolated from uninfected individuals. In contrast, comparison of CpG- or R848-induced cytokine production by pDC from the two groups revealed no differences, including the classical pDC derived cytokines IFN-α and CXCL-10. Taken together, these data show that mDC but not pDC from *S. haematobium-*infected individuals are functionally impaired in their responses to TLR ligands tested here in terms of upregulation of maturation markers and cytokine expression.

### Reduced TLR4 responses of mDC coincide with reduced pro-inflammatory MAPK signaling to LPS, but not with altered TLR4 expression

**Figure 3:**
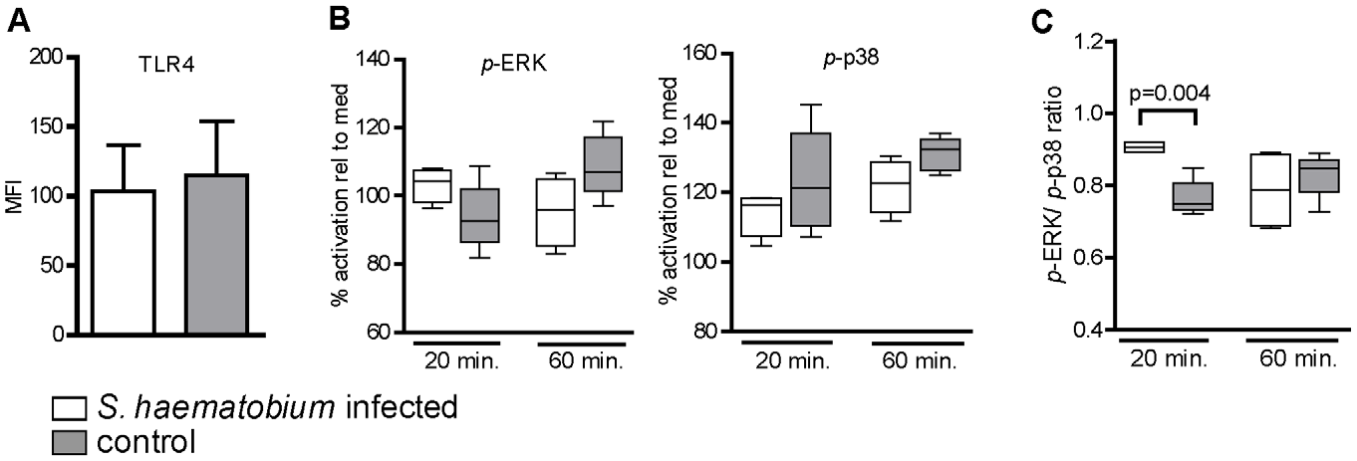
mDC from *S. haematobium*-infected subjects have an altered MAPK signaling, but not TLR4 expression profile. (A) TLR4 expression was analysed on mDC present in PBMC following the gating strategy as described in legend of Fig. 1. (B and C) 20 and 60 minutes after stimulation with LPS, mDC were intracellularly stained for (B) phospophorylated p38 and ERK and (C) the ratio between *p*-ERK and *p*-p38 was determined by dividing the respective mean fluorescence intensities per sample. Each group represents data from 5 donors. (A) Bars represent mean + SD. (B and C) box plots represent 25–75 percentile range with error bars showing minimum to maximum.

To explore the mechanisms underlying the reduced TLR responsiveness in mDC from infected subjects, we focused on LPS as a stimulus since triggering of TLR4 by LPS is one of the most well characterized pathways leading to myeloid DC activation [21]. First TLR4 expression on mDC was analysed. However, no differences were seen in surface expression of TLR4 on mDC between both groups (fig. 3A), suggesting that other mechanisms underlie the observed differences in TLR4 responsiveness. TLR-mediated DC activation is known to be mediated by signaling molecules that include the mitogen activated protein kinases (MAPK) p38 and ERK. Activation of p38 downstream of TLR triggering in DC has been shown to be important for expression of maturation markers and cytokines production, whereas ERK phosphorylation is linked to dampening of these responses [22]. Given that schistosomal antigen preparations have been shown to modify TLR responses through modulation of these MAPK [19], [20], [23] we asked whether altered TLR signaling via p38 and ERK could provide an explanation for the reduced TLR responsiveness in the infected group. Phosphorylation of MAPK p38 and ERK was determined at 20 and 60 minutes after TLR4 stimulation, timepoints at which the activation of these MAPK in DC are known to peak following TLR engagement [20], [23], [24]. We found that stimulation of mDC by LPS resulted in a higher phosphorylated ERK/p38 ratio in the *Schistosoma*-infected group compared to the controls (Fig. 3C), which was due to a tendency towards higher ERK phosphorylation on the one hand and lower p38 phosphorylation on other hand (Fig. 3B). These data show that mDC from infected individuals display a reduced induction of pro-inflammatory MAPK activation in response to LPS. These results indicate that a change in downstream signaling, rather than TLR4 expression itself, may be responsible for the impaired responsiveness of mDC from *S. haematobium* infected individuals towards LPS.

### mDC from S. *haematobium* infected subjects have impaired T cell activation capacity, due to reduced HLA-DR expression

**Figure 4:**
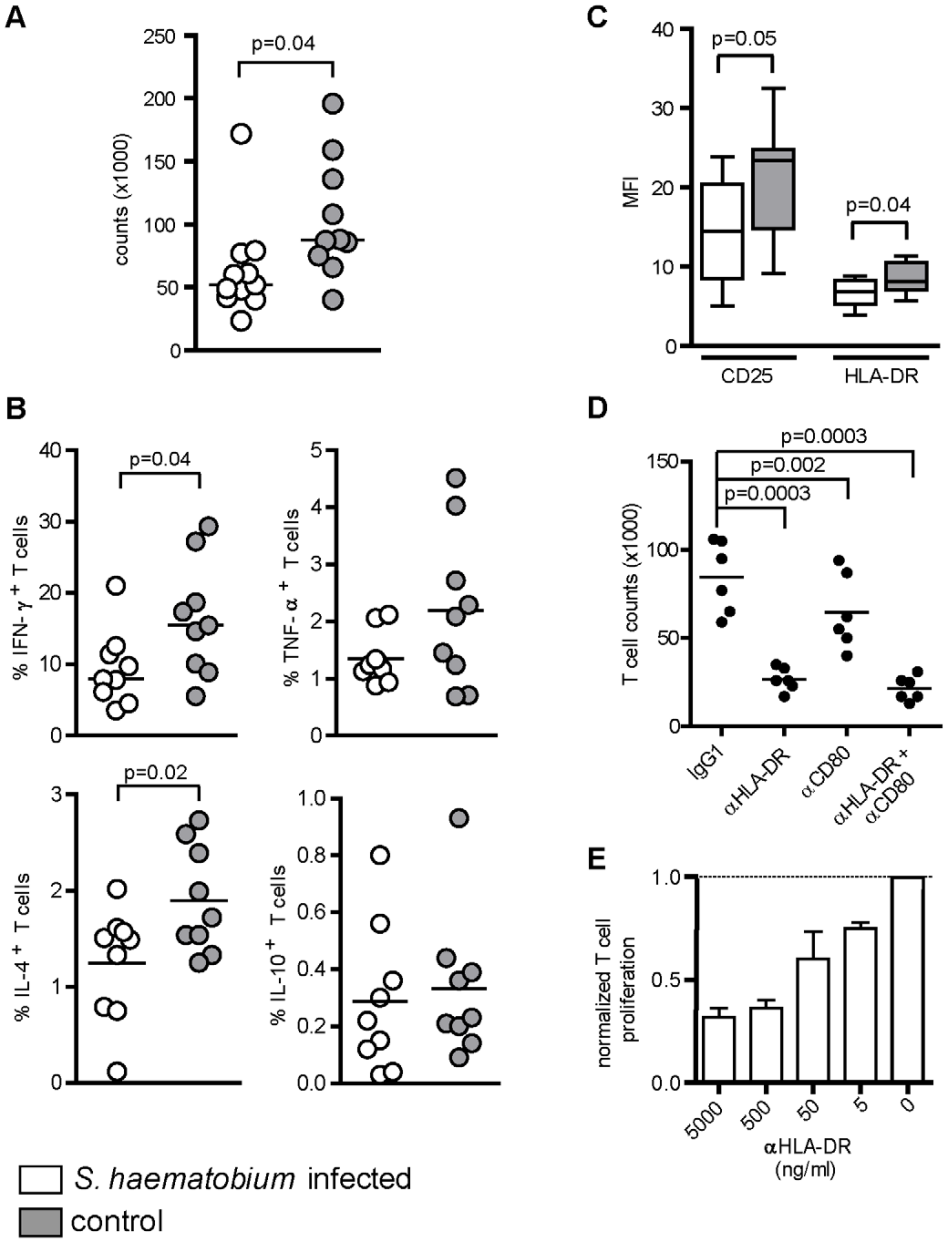
mDC from *S. haematobium*-infected subjects have a reduced T cell activating capacity due to lower HLA-DR expression. LPS matured mDC were cocultured with allogeneic naïve T helper cells for 6 d after which (A) T cell expansion was determined with a counting chamber and (B) Intracellular cytokine production or (C) CD25 and HLA-DR expression was assayed by FACS, 6 h after restimulation with phorbol 12-myristate 13-acetate (PMA) and ionomycin in the presence of brefeldin A for the last 2 h. (D+E) LPS-matured mDC from European controls were cocultured with allogeneic naïve T helper cells for 6 d in the presence of depicted neutralizing antibodies and T cells were counted as in (A). T cell counts are shown as (D) absolute values or (E) relative to the control condition. (A,B,D) Horizontal bars represent mean based on data from 9 donors in each group. (C) Box plots represent 25–75 percentile range with error bars showing minimum to maximum based data of 9 donors in each group. (E) Bars represent mean + SD of 3 independent experiments.

DC are instrumental in the priming and regulation of T cell responses. In this process antigen presentation, costimulation and cytokine production by DC play a crucial role. Since these processes were affected in mDC from *S. haematobium*-infected subjects, we assessed the T cell-priming capacities of these DC. To address this, allogeneic naïve T helper cells were cocultured together with LPS-stimulated mDC. After 6 days T cell expansion and T cell cytokines were analysed. Interestingly, T cells primed by mDC from *Schistosoma*-infected subjects had expanded significantly less than the T cells in the presence of mDC from uninfected subjects (Fig. 4A). Schistosomes are known to condition DC to promote Th2 and regulatory type T cell responses [2], [9], characterized by an increased production of IL-4, IL-5, IL-13 and IL-10, respectively. Yet, total cytokine production in day 6 culture supernatants (data not shown) or intracellular cytokines following polyclonal restimulation (Fig. 4B) revealed no differences in T cell polarization by mDC from infected or uninfected individuals. However, the frequency of IL-4 or IFN-γ producing T cells was significantly lower when primed by mDC from infected subjects compared to uninfected individuals. In contrast, the proportion of IL-10 or TNF-α secreting T cells was unchanged (Fig. 4B). In addition, these T cells expressed lower levels of the activation markers HLA-DR and CD25 [25] (Fig. 4C). This suggests, that mDC from *S. haematobium* infected subjects do not drive distinct polarized effector T helper cell responses, but seem to have a general impairment to induce T cell activation and effector T cell expansion.

For proper T helper cell activation and expansion, TCR triggering (signal 1) and costimulation (signal 2) are provided by DC through peptides/MHC class II complexes and costimulatory molecules CD80 and CD86, respectively [26], [27]. Since HLA-DR and CD80 surface expression on mDC from infected individuals following TLR stimulation tended to be lower compared to levels found on mDC from uninfected subjects (Fig. 2B), we assessed whether blocking of HLA-DR and/or CD80 on control mDC could mimic their functional impairment to activate T cells. Indeed, neutralization of HLA-DR during the DC-T cell cocultures strongly suppressed the T cell activating capacity of mDC as determined by T cell proliferation (Fig. 4D) and cytokine production (data not shown). In contrast, complete blocking of CD80 only minimally inhibited T cell expansion (Fig. 4D). This observation, together with the fact that only minimal concentrations of HLA-DR blocking antibody could already interfere with mDC induced T cell activation (Fig. 4E), point in the direction that the observed differences in HLA-DR expression, although small, play a major role in the different capacity of the mDC from infected and uninfected subjects to drive T cell activation.

## Discussion

There is a wealth of evidence from both *in vitro* and murine models that helminth parasites or their secreted products are able to modulate and suppress DC function [28], [29]. However, apart from a recent report that has documented a suppressed function of *in vitro* generated monocyte-derived DC isolated from hookworm infected subjects [30], there is currently little known about the actual consequences of chronic helminth infections on human DC function *in vivo*. In the study presented here, we now provide support for the notion that human blood DC are functionally impaired during chronic helminth infection *in vivo*, by showing that circulating mDC isolated from *Schistosoma*-infected subjects have an reduced capacity to respond to TLR ligands and to initiate T helper cell responses.

Quantification of circulating mDC and pDC in blood from infected subjects revealed that these cells are present in reduced frequencies as well as numbers/ml blood compared to controls. This appears to be a phenomenon that is associated with more chronic infections in general, since similar observations have been made in patients with chronic Hepatitis B [31], HIV [32], tuberculosis [33] and malaria infection [34]. The reason for lower frequencies remains unclear, but similar to what has been documented for microfilaria of the species *Brugia malayi* [35], [36], *Schistosoma* parasites may promote direct apoptosis in DC. An increase in migration out of the circulation would also be a possible explanation, since chronic *Schistosoma* infection may favor migration of DC into inflamed tissues or lymphoid organs. Finally, the output of DC from the bone marrow could be altered, since we also observed lower monocyte, but not T cell frequencies in the circulation of the infected individuals (unpublished data), a cell type closely related to mDC and originating from same bone marrow precursors [37], [38].

In order to exert their function as innate immune cells, DC have to be able to sense and respond rapidly to external stimuli, including pathogen derived components. This is reflected in the release of cytokines and expression of a set of membrane bound molecules crucial for the initiation of the adaptive arm of the immune response [27]. We found that mDC from infected subjects displayed reduced responses towards TLR ligands LPS and R848. The fact that all analysed cytokines, including IL-10, were lower expressed in response to R848 by mDC from infected subjects compared to uninfected controls, suggests that these DC don't have a more anti-inflammatory phenotype but instead have a general impairment respond to TLR ligation. Furthermore, the selective impairment to upregulate HLA-DR by mDC from infected subjects was found after stimulation with both LPS and R848. This suggests that the impaired response may not be TLR-specific but rather a more general phenomenon. Impaired TLR responsiveness of blood DC has been reported during other chronic infections as well, where both reduced TLR expression [39], [40] as well as altered signaling [40], [41] has been implicated as underlying mechanisms. We could not observe any difference in TLR4 expression between the two groups. However, we did find a tendency towards lower p38 activation in mDC from the infected group when compared to the uninfected group in response to LPS. The fact that p38 activation in DC is important for mediating pro-inflammatory signaling following triggering of multiple TLR [42], may point in the direction that a general deficit of pro-inflammatory signaling, rather than TLR expression itself, underlies the impaired responsiveness of mDC towards TLR ligands from *S. haematobium* infected individuals.

In addition to defects in innate responses of the DC, we observed an impaired capacity of these cells to prime T cell responses. Given that chronic *Schistosoma* infection is generally associated with a Th2-like as well as anti-inflammatory immunological profile [1], [2] and several *in vitro* studies have shown that schistosomal antigens can drive these responses via DC [18], [20], [43]–[45], one could hypothesize that blood DC from infected individuals would favor the differentiation of naïve T cell towards these subsets. However, we observed a general failure to prime and activate a T helper cell response by mDC, reflected by reduced T cell expansion and a general impairment in production of both Th1 and Th2 associated cytokines after restimulation. An explanation for this discrepancy may be provided by the current view that only after migration into tissues blood DC receive the right differentiation signals to acquire their full T cell polarizing potential [46]. Alternatively, the level of potentially polarizing factors in blood may be too low to affect the polarizing capacity of these DC.

With regard to the mechanisms that may underlie the impaired capacity of these DC to prime T cell responses, we found a tendency towards lower HLA-DR as well as CD80 expression on LPS-matured mDC derived from infected individuals. Both TCR triggering and costimulation are instrumental for DC-mediated T cell activation [27]. However, the fact that even partial neutralization of HLA-DR in mDC – T cell cocultures had a similar impact on T cell activation as total blockade of CD80, makes it more likely that primarily the reduced HLA-DR expression, though small, accounts for the observed differences in T cell-activating capacity. Nonetheless, involvement of other membrane-bound or soluble factors in the observed differences in T cell priming cannot be excluded. A recent *in vivo* murine study has shown that *Schistosoma* infection can lead to T cell anergy through induction of PD-L1 on macrophages [13]. However, we did not find any differences in expression of PD-L1 between the mDC of the two groups (data not shown), making a role for PD-L1 in the observed differences unlikely. Thus, we conclude that probably not an increase in negative costimulatory signals, but most likely a lack of antigen presentation underlies the reduced capacity of blood DC from infected individuals to prime a T cell response.

It remains to be established what the mechanisms are that underlie the impaired DC function in *Schistosoma*-infected individuals. Modulation of DC function by direct interactions with *Schistosoma*-derived antigens would provide a plausible explanation. Several studies have documented potent modulatory effects of schistosomal antigens on the function of *in vitro* generated DC, that include suppression of TLR-induced cytokine production, maturation marker expression and MAPK signaling [19], [20], [47] and manipulation of DC-driven T cell responses [13], [18], [24], [48]. Adult *S. haematobium* worms are found in the venous plexuses around the urinary bladder, thereby providing a physiological situation in which circulating DC could constantly be exposed to potentially modulatory antigens released by these parasites in the blood. In this respect, an interesting finding was that mDC from infected subjects, but not pDC, were less responsive to TLR ligands. Schistosomal antigens can be recognized by and have been shown to exert their modulatory effects on DC through engagement of certain TLRs, such as TLR2 [18], [20] and c-type lectins like DC-SIGN [47], [49] which are preferentially expressed by myeloid, and not the plasmacytoid DC lineage [50], [51]. Apart from this direct mechanism, chronic *Schistosoma* infection may also lead to attenuation of DC function indirectly by induction of regulatory immune responses. For instance, important regulatory cytokines produced by the host, such as IL-10, have the potential to suppress DC function [15] and to be elevated in *S. haematobium* infected subjects [17]. Lastly, chronic schistosomiasis has also been shown in several studies to negatively affect the nutritional status of the host [52], [53]. Although there is currently little known about the consequences of malnutrition on the functionality of DC during schistosomiasis, experimental models show that undernutrition as such can lead to an impaired function of APC resulting in a diminished induction of adaptive immune responses [54], [55].

Despite the fact the infected and healthy individuals enrolled in this study were sex and age matched and living in the same endemic area (Table 1), we cannot formally exclude that other environmental factors, such as co-infections, could contribute to observed differences in DC phenotype and function. In this respect, since filarial and malarial infections are also highly endemic in Gabon and have been documented to modulate APC function [40], [56], [57], the participants in this study were screened for infection by these parasites. While only a single individual was tested positive for malaria infection, several cases of filariasis were found in both groups (Table 1). However, stratification based on filarial infection revealed that these parasitic infections could not account for the observed differences in DC function (data not shown). Furthermore, other endemic chronic infections such as HIV, hepatitis and tuberculosis, are not likely to underlie the selective dysfunction of mDC either, since in contrast to our findings, these types of infections have been found to be associated with functional impairment of pDC [33], [39], [58], [59].

In summary, the data presented in this study provide support for the notion that chronic schistosomiasis results in suppression of human DC function *in vivo*. This sheds new light on the mechanisms that could underlie the immune hyporesponsiveness observed during chronic helminth infections [2], [14]. Importantly, a general impairment in TLR responses of mDC and subsequent priming of T cell responses, may not only lead to impediment of immune responses against the worms, but may also have consequences for proper induction of protective immunity against concurrent infections, especially from bacterial or viral origin, which draws heavily on TLR-driven APC activation [60]. Finally, suppressed function of DC, as we documented here during a chronic helminth infection, has also been documented in chronic infections caused by other pathogens such as bacteria [33] or viruses [31], [58], [59]. This suggests that an impaired DC function during persistent infections is a widely distributed phenomenon and points in the direction that targeting of DC is a common strategy evolved by pathogens to subvert immune responses and establish chronic infections.
